# Importance of the Working Environment for Early Retirement: Prospective Cohort Study with Register Follow-Up

**DOI:** 10.3390/ijerph18189817

**Published:** 2021-09-17

**Authors:** Emil Sundstrup, Sannie V. Thorsen, Reiner Rugulies, Mona Larsen, Kristina Thomassen, Lars L. Andersen

**Affiliations:** 1 National Research Centre for the Working Environment, 2100 Copenhagen, Denmark; SVT@nfa.dk (S.V.T.); RER@nfa.dk (R.R.); KRT@nfa.dk (K.T.); LLA@nfa.dk (L.L.A.); 2 Department of Public Health, University of Copenhagen, 1014 Copenhagen, Denmark; 3 Department of Psychology, University of Copenhagen, 1353 Copenhagen, Denmark; 4 The Danish Center for Social Science Research, 1052 Copenhagen, Denmark; ML@vive.dk

**Keywords:** health, older worker, physical work demand, physical workload, psychosocial, retirement, statutory retirement age

## Abstract

Background: This study investigates the role of physical work demands and psychosocial work factors for early retirement among older workers. Methods: Data from three Danish surveys on work environment and health among employed older workers (age 55–59) were merged with a national register containing information on labour market participation. Robust Poisson regression modelled the risk ratios (RR) and 95% confidence intervals (CI) for the association between physical and psychosocial work factors and early retirement, that is, not working after the age of 64. Results: Of the 2800 workers, 53% retired early. High physical work demands (RR 1.33, 95% CI 1.19–1.48), poor overall psychosocial working conditions (RR 1.43, 95% CI 1.26–1.61), and access to early retirement benefits (RR 1.79, 95% CI 1.53–2.10) predicted early retirement. Subgroup analyses revealed that poor overall psychosocial working conditions were a stronger predictor for early retirement among workers with seated jobs than those with physically active jobs. Conclusions: High physical work demands and poor psychosocial working conditions are factors that can push older workers out of the labour market prematurely. Poor psychosocial working conditions seem to be a particularly strong push factor among workers with seated work.

## 1. Introduction

Demographic changes, which are the net outcome of a fairly low level of fertility and an increase in longevity, have led to increasing calls for longer working lives. Thus in many countries, it has become a political priority to keep older workers longer in the labour market and several policies have been implemented to achieve this goal [1]. Consequently, the official state pension age is increasing in many countries worldwide and economic incentives for early voluntary retirement have been reduced. In Denmark, a general principle has been established that the state pension age increases with increases in life expectancy, based on the assumption that Danes should have an average of 14.5 living years after retirement [2]. Thus, the state pension age has increased from 65 years in 2018 to 66.5 years in 2021 and is set to increase even further to 70 years in 2040 [3]. Importantly, the employment rate of older Danish workers (aged 60–64) is higher than the OECD average (60.3% versus 49.6%), but it is still lower than in other Scandinavian countries such as Sweden (70.2%) and Norway (64.4%) [1,4]. Thus, there seems to be potential to further increase labour market participation in Denmark. However, a proportion of older workers may lack the resources to sustain an extended working life with prolonged physical and psychosocial exposures, and there are still many people in Denmark who—voluntarily or involuntarily—leave the labour market before the official state pension age.

Many factors can influence the complex and dynamic decision to leave the labour market. Factors stimulating older workers to leave the labour market before the official state pension age are known as push, pull, and jump mechanisms [5,6]. Push refers to involuntary labour market exit where people are being ‘pushed’ out, for example, due to adverse physical and psychosocial working conditions or poor health. Pull is defined as voluntary early retirement initiated by attractive early retirement schemes or norms and conventions on when it is appropriate to withdraw from employment. Jump refers to voluntary early retirement triggered by personal needs and values, for example, spending time with grandchildren or the desire to travel the world [7]. Largely, push factors seem more modifiable on a workplace level than pull and jump factors, which are more related to financial incentives [7].

High physical work demands are a well-known push factor and previous studies have found them to associate with the risk of sickness absence [8,9,10,11], early retirement [12,13,14], and disability pension [14,15,16] especially among older workers [17]. For instance, results from the Helsinki Health Study cohort showed that physical work demands were among the primary risk factors for all-cause disability pension [15]. Further, years of exposure to physical work demands during working life have been shown to increase the risk of disability pension and sickness absence in a dose-response fashion [14]. Age influences the association between physical work demands and health, and older workers are especially at increased risk for a disability pension from high physical work demands [5,18].

Adverse psychosocial working conditions can also act as push factors, evidenced by studies reporting that low job control [15,19,20,21] and low skill discretion [13] are predictive of early labour market exit. A systematic review, including a meta-analysis, assessing the contribution of psychosocial work factors to disability pension found moderate evidence for the role of low job control and the combination of high demands and low control (job strain) as predictors for disability pension [22]. Since the majority of studies investigating the influence of psychosocial working conditions on early labour market exit are limited to the job strain model, the review recommended the measurement of specific exposure factors in future studies [22].

Even though both physical and psychosocial work factors are well-known risk factors for poor health and early labour market exit, knowledge on the interplay between these work factors is scarce. Specifically, knowledge on whether the relation between specific psychosocial work factors and early labour market exit differs by physical work characteristics is lacking. Health has been shown to be an important factor for leaving and staying, and the occupational consequences of poor physical health have been shown to be higher among workers with physically demanding work [23]. In a recent cross-sectional study, senior workers were asked about possible reasons for staying longer at the labour market, and those with physically demanding work would do so if the work were less physically strenuous [5]. By contrast, those with mainly seated work stated that they would stay longer if the work were less mentally demanding and if they got more influence on planning the work [5]. Thus, to improve our knowledge about how to direct efforts at the workplace towards preventing early retirement among workers with different types of jobs, we investigate—in a prospective cohort design—if the relative importance of the psychosocial working environment for early retirement differ between senior workers with physically and sedentary job tasks. If the psychosocial work environment is more important for senior workers with sedentary work, we would expect to find a significant interaction between the psychosocial work environment and a measure for sedentary/physical work in our statistical model.

In Denmark, the voluntary early retirement program (VERP) is a scheme for people who are members of an unemployment insurance fund and have paid retirement contributions for at least 30 years, which allows for withdrawal from the labour market before the official state pension age. VERP has been considered a factor that, due to financial incentives of an attractive retirement scheme, pulls people out of the labour market. However, it has been suggested, that older workers with declining health may choose to wait for early retirement benefits instead of applying for a disability pension [24,25]. Thus, VERP could be seen as a way out of the labour market if the push factors are strong, for example, exposure to physically demanding works and/or adverse psychosocial working conditions. This is in agreement with previous Nordic studies showing that early voluntary retirement schemes are utilised to a higher extent among those with physically demanding work [26]. Further, calculations by Bingley et al. found such programs to be more attractive for low-income than high-income workers [27].

The aim of the study was first to investigate the role of physical and multiple psychosocial work factors for early retirement defined as not working after the age of 64. Further, distinguishing between workers with physically active jobs and seated jobs, the second aim was to investigate whether the association of psychosocial work factors and access to early retirement benefits on early retirement differed by physical work characteristics. These analyses can help guide preventative strategies aiming at reducing risk factors for early labour market exit and, thereby, facilitate sustainable employment among both workers with physical and workers with sedentary job tasks. The novelty of the paper is that separate but symmetric analyses are conducted on samples of older workers with physically active jobs and seated jobs, respectively. This provides comparable estimates of the relative importance of several psychosocial work factors and access to early retirement benefits on the risk of early retirement for these two groups of workers.

## 2. Materials and Methods

### 2.1. Study Design

This prospective cohort study combines data from three work environments and health surveys in Denmark (DWECS 2005 and 2010 and DANES 2008) with information about labour market participation from the ‘Danish Labour Market Accountant register’ (LMA) (Danish: Arbejdsmarkedsregnskab uden timenormering).

### 2.2. Study Population

DWECS 2005 (*N* = 19 855) and DWECS 2010 (*N* = 31 210) were performed in the general working population. DANES 2008 (*N* = 9913) were performed in the general working population but also included an additional oversampling of those aged ≥50 years (*N* = 4477, the sub-sample is called DANES 2008-senior). The response percentages of DWECS 2005 and 2010 and DANES 2008 were 63%, 53%, and 76%, respectively.

For the present study, we used the following inclusion criteria, where each step adds to the previous criteria: (i) age 55–59 years and responding to the questionnaire (*N* = 6079), (ii) actively employed at the time of the questionnaire response (*N* = 5253), (iii) alive at age 65 (*N* = 5097), (iv) 65 years or older in 2018, that is, each respondent was followed in the LMA register until they turned 65 years (*N* = 3787), (v) we excluded 3 individuals under education (*N* = 3784), (vi) 733 individuals with missing covariates (*N* = 3051), and (vii) 251 individuals with missing predictor variables (*N* = 2800). Baseline characteristics of the study population of 2800 aged 55–59 at baseline can be seen in Table 1.

Data from the present study regarding work factors facilitating work beyond state pension age has previously been published [28].

### 2.3. Predictors

The present study examined to which extent access to early retirement benefits and eight different work environment factors predicted early retirement.

Physical work demands were assessed with the single-item question *“How would you describe your physical activity at your main job?”* with four response categories (1) Mostly sedentary work that does not require strenuous physical activity; (2) Mostly work while standing or walking but does not require strenuous physical activity; (3) Work while standing or walking with some lifting and carrying; (4) Heavy or fast moving work that is physically strenuous [29]. For further analyses, the physical work demands factor was linearly normalised on a scale of 0 to 1, that is, 0 = ’seated work’, 1/3 = ’standing and walking at work’, 2/3 = ‘lifting and carrying’, and 1 = ‘heavy and fast’ [28].

For the psychosocial work factors, seven questions that were available in all three cohorts were included. All questions were originally developed for the Copenhagen Psychosocial Questionnaire (COPSOQ II) [30,31] and included (i) influence at work (*“Do you have a large degree of influence concerning your work?”*), (ii) work pace (*“Do you have to work very fast?”*), (iii) time to complete tasks (*“How often do you not have time to complete all your work tasks?”*), (iv) received information about decisions at work (*“At your place at work, are you informed well in advance concerning, for example, important decisions, changes, or plans for the future?”*), (v) received information needed to do work well (*“Do you receive all the information you need in order to do your work well?”*), (vi) recognition from management (*“Is your work recognized and appreciated by the management?”*), and (vii) possibilities for development (*“Do you have the possibility of learning new things through your work?”*). The response categories were “Always”, “Often”, “Sometimes”, “Rarely”, “Never/almost never” for psychosocial factor (i), (ii), and (iii), and response categories were “To a very high degree”, “To a high degree”, “To some degree”, “To a slight degree”, and “To a very slight degree” for psychosocial factor (iv), (v), (vi), and (vii). For further analyses, the response categories of the psychosocial variables were linearly normalized on a scale of 0–1, where 0 is best and 1 is worst [28].

We also created a combined psychosocial work environment factor. We first dichotomised all seven psychosocial variables with a cut point between “Often” and “Sometimes”, for psychosocial factor (i) influence, (ii) work pace, (iii) time to tasks, and between ‘to a high degree’ and ‘to some degree’ for (iv) information about decisions, (v) information to do well, (vi) recognition from management, (vii) possibilities for development. Note (ii) ‘work pace’ and (iii) ‘time to task’ were turned around in the scoring, so poor work environment always had a high score. We then calculated a combined score for all seven questions by taking the average of the dichotomised variables [28].

### 2.4. The Danish Early Retirement Scheme

Access to the Danish voluntary early retirement program (VERP) is an important economic factor that may pull older workers out of the labour market. The VERP is partly paid by the state and partly by the employees’ own savings. To have access to early retirement benefits the employee must have paid to the scheme for at least 30 years. For individuals in our sample, VERP benefits were available from age 60 to 64 (after which state pension is given and other rules apply). The employee could receive higher VERP benefits if he/she waited to begin receiving these benefits at age 62 or later.

### 2.5. Outcome

The outcome variable is ‘working at age 64 to 65′ (yes/no), that is, we considered people to have retired early from the labour market, willing or unwilling, if they did not have any work hours according to the LMA register after the age of 64. The register contains individual day-to-day information about labour market participation, unemployment, education, granted social benefits, etc. of all citizens in Denmark [32]. With this definition, 53% of our study population had retired early at age 64.

### 2.6. Covariates

In the analyses, we adjusted for sex (man, woman), age at baseline (continuous variable), cohort (DWECS2005, DWECS2010, DANES2008), cohabiting (married, cohabiting, single), sector (public sector, private sector), body mass index (BMI: underweight < 18.5, 18.5 ≤ normal weight < 25, 25 ≤ overweight < 30, 30 < obese), smoking status (never, ex-smoker, smoker), household disposable income (after tax) (0–15 percentile, 15–50 percentile, 50–85 percentile, 85–100 percentile), weekly working hours (≤35 h, 35–40 h, >40 h), vocational education (unskilled, skilled, and higher education), and long-term sickness absence within 2 years before baseline (yes, no). Furthermore, we mutually adjusted for the physical work environment, the combined factor for the psychosocial work environment, and the variable measuring access to VERP. The individual psychosocial work environment factors were each adjusted for the physical work environment and ‘access to VERP benefits’.

Information about sex, age, cohabitation, sector, family available income, vocational education, and previous long-term sickness absence was obtained at each respective baseline (2005, 2008, and 2010) based on registers from Statistics Denmark. Bodyweight and height (to subsequently calculate BMI), as well as smoking status and work hours, were self-reported in the questionnaires.

### 2.7. Statistics

We used robust Poisson regression analysis (Proc Genmod, SAS version 9.4, SAS institute Inc. Cary, NC, USA), even though the outcome could only take the values ‘0′ and ‘1′, in which case one usually would use logistic regression. An assumption for the Poisson regression is that the mean of the outcome is approximately equal to the variance in data. In our data, the mean (of working/not working) is equal to 0.53 and the variance is 0.25, hence our data are under-dispersed. This is related to the outcome only can take the values ‘0′ and ‘1′. To compensate for this, we used robust Poisson (with the robust sandwich variance estimator) regression. Robust Poisson regression increases the confidence intervals of the estimated results. We chose to use robust Poisson regression because it is preferable above logistic regression when the outcome is common (i.e., >10%) since the risk ratios from Poisson regression are more intuitively understandable [33].

We modelled the risk ratio for early retirement from the labour market before the age of 64. In model 1 (minimally adjusted), we only adjusted for sex, baseline age, and cohort. In model 2 (fully adjusted), we adjusted for all covariates mentioned before, including access to VERP. Because sickness absence may be on the causal pathway from exposure (physical and psychosocial work factors) to outcome (early retirement), controlling the analyses for previous LTSA could be an over-adjustment. Thus, a sensitivity analysis was performed without adjusting the final Model 2 for the previous LTSA.

We calculated multiplicative interaction terms between physical work demands and each of the psychosocial work factors by including both the psychosocial factor and the physical activity at work factor in the same fully adjusted analysis, plus an interaction term between the particular psychosocial factor and the physical work demands. For this purpose, the question about physical work demands was dichotomized, with ‘physically active work’ defined as the last three response categories, and ‘seated work’ defined as the first response category.

## 3. Results

Table 1 shows the baseline characteristics of the study sample. Of the 2800 workers aged 55–59 at baseline, 1478 (53%) had retired early from the labour market at age 64.

Table 2 shows that physically demanding work in the fully adjusted model was associated with early retirement (RR 1.33, 95% CI 1.19–1.48). We obtain a similar result for all subgroups when stratifying the analysis by education and gender, respectively, although the association seems to be most pronounced among those with higher education and among men, see Supplementary Material. In other words, having a physically demanding job seems mainly to make a difference within the group of higher educated and within the group of men, when deciding whether or not to retire early.

When combining the seven psychosocial work factors, poor overall psychosocial working conditions were associated with early retirement (RR 1.43, 95% CI 1.26–1.61). In the fully adjusted model, six of the seven psychosocial working conditions were significantly associated with early retirement: low influence at work, no time to complete tasks, no information about decisions, no information to do work well, no recognition from management, and no possibilities for development (RR range of 1.19–1.41). Table 2 also shows, that having access to VERP was associated with early retirement.

Table 3 shows that the interaction term between physical work (Yes/No) and overall psychosocial working conditions was statistically significant (*p* = 0.0039). Further, the interaction term between physical work and access to VERP benefits was statistically significant (*p* = 0.0487). Thus, overall psychosocial working conditions were a stronger predictor among those with seated work than among those with physically demanding work, while the opposite is found when it comes to access to VERP benefits. The latter result is in line with results of stratified analyses in Supplementary Material suggesting that the association between early retirement and access to VERP benefits is more pronounced, the lower the level of education. By contrast, the association between early retirement and psychosocial working conditions does not seem to depend significantly on education. However, this association seems to be stronger for men than for women. In other words, men seem to be more affected by poor overall psychosocial working conditions than women when deciding whether or not to retire early.

### Sensitivity Analysis

The sensitivity analysis (i.e., not adjusting the final Model 2 for previous LTSA) revealed only minor changes to some of the risk estimates and did not change the results to any statistically significant extent.

## 4. Discussion

High physical work demands, poor psychosocial work environment, and access to voluntary early retirement benefits predicted early retirement among older workers. Within the psychosocial working environment, low influence at work, no time to complete tasks, no information about decisions, no information to do work well, no recognition from management, and no possibilities for development predicted early retirement. Subgroup analyses of those with seated and physically active jobs did not change the overall picture, however, poor overall psychosocial working environment was a stronger predictor for early retirement among those with seated work than among those with physically demanding work.

### 4.1. Physical Work Demands

High physical work demands predicted early retirement and can, therefore, be considered a push factor. This is in agreement with previous studies documenting that high physical work demands increase the risk of prematurely leaving the labour market through early voluntary retirement programs [12,13,14] or disability pensions [14,15,16]. In line with this, Andersen et al. found that higher physical work demands decreased the likelihood of working beyond state pension age, further establishing that physical demands act as a push factor [28]. In a workplace setting, reduced exposure time to physical work demands—such as heavy lifting, pulling, and bending could, for example, be achieved by organizing the work differently (e.g., by incorporating micro-breaks or job rotation to less physically demanding job tasks) or by using technical aids, for example, lifting devices, when appropriate. Thus, adjusting the physical work demands to the capacity of older workers may be a strategy to reduce push from physical work, and thereby support the employment of older workers.

### 4.2. Psychosocial Working Conditions

Our results show that poor psychosocial working conditions act as a push factor through its association with early retirement. This is in agreement with previous studies showing that adverse psychosocial working conditions increase the risk of early labour market exit [13,15,19,20,21,22,34,35]. For instance, a study by Leijten et al. showed that higher autonomy, higher support, and lower psychosocial job demands diminished the risk of disability pension among workers with poor health by 82%, 49%, and 11%, respectively [36]. In line with this, Andersen et al. reported that higher influence, lower work pace, more time to complete tasks, more information about decisions, more information to do work well, higher level of recognition from management, and better possibilities for development increased the likelihood for working beyond retirement age [28]. In a systematic review, Knardahl et al. found moderate evidence for the role of low job control, and for the combination of high demands and low control (job strain) as predictors for disability pension [22].

As the most important finding, the present results indicate that a poor overall psychosocial work environment is a stronger push factor among workers with seated work than among those with physically demanding work. Even though a poor psychosocial work environment acted as a push factor among both employees with seated and physically active jobs, the interaction analysis revealed that it was a stronger predictor for early retirement among those with seated work. The results indicate that efforts at the workplace aiming at improving psychosocial working conditions could have a larger impact on preventing early labour market exit among older workers with mainly seated work. Importantly, improving psychosocial working conditions among older workers with physically demanding jobs still seem of great importance for promoting work participation and should be implemented in parallel with interventions aiming at reducing any unnecessary physical work demands. By contrast, a previous study about working beyond state pension age by Andersen et al. did not find the interaction term between physical activity at work and psychosocial work environment to be statistically significant for any of their included psychosocial variables [28].

### 4.3. Voluntary Early Retirement Program (VERP)

Access to early retirement benefits through the VERP predicted early retirement, both for those with physically demanding and seated work. VERP has been considered a factor that pulls people out of the labour market, but it may also be a way out of the labour market for those experiencing strong push factors, for example, exposure to physically demanding work and/or adverse psychosocial working conditions. In line with this, it has previously been suggested that senior workers (50–60 years) with deteriorating health may choose to wait for VERP benefits instead of applying for disability pension [24,25]. The results also indicate, that being a member of VERP can, to a higher degree, be used to predict early retirement among those with physically demanding work compared to seated work. This was acknowledged by the interaction analysis, indicating that enrolment in VERP is a somewhat stronger predictor among those with physically demanding work. This is in agreement with a previous prospective cohort study among senior workers in Denmark showing that physically demanding work during working life is a predictor for leaving the labour market through VERP [14]. Thus, it seems plausible that both pull and push factors act when workers choose to prematurely leave the labour market through the VERP. Further, being a member of the voluntary early retirement program increased the chance of early retirement, especially for those with shorter education. This could express a stronger desire to leave the labour market—when possible—among those with shorter education. The association between psychosocial working conditions and early retirement was, to a lesser extent, dependent on education, except for information to do the work well, which was more influential among those with longer educations. Thus, workplaces should ensure a good psychosocial working environment regardless of the length of education of the workers.

### 4.4. Strengths and Limitations

A strength of the study is the prospective study design with register follow-up. Specifically, the use of register-based data on labour market participation derived from the high reliable LMA register is a strength [32]. All transfer payments are systematically recorded on a daily basis in LMA, which enables the assessment of labour market outcomes free from potential self-reporting bias.

Another strength is that we conducted separate but symmetric analyses on samples of older workers with physically active jobs and seated jobs, respectively, providing for the first time, comparable estimates of the relative importance of a number of psychosocial work factors and access to early retirement benefits on the risk of early retirement for these two groups of workers.

The analyses were controlled for several factors that may influence the decision to retire early from the labour market [9], for example, sex, age, cohabitation, sector, family available income, vocational education, and previous long-term sickness absence. Some of the control variables may be confounders whereas others may be effect modifiers. Having only one measurement point at baseline, it cannot be determined whether they are mediators or not (i.e., on the causal pathway to early retirement). In line with this, it could be argued that controlling for previous long-term sickness absence is an over-adjustment, since sickness absence may be on the causal pathway from exposure (physical and psychosocial work factors) to outcome (early retirement). To test this, we performed a sensitivity analysis without adjusting the final model 2 for the previous LTSA. We observed only minor changes to some of the risk estimates and it did not change the results to any significant extent. Thus, the estimates in the fully adjusted model 2 form the base for all work-related outcomes for the discussion, although the reader should be aware of the possible bias associated with over-adjustment in the analyses.

A limitation is that information on physical and psychosocial working conditions assessed through questionnaire surveys depends on participants’ memory, understanding, and interpretation [37,38]. A limitation of the study is, therefore, that information on the working environment can be affected by self-reporting bias. On the other hand, it is a strength, that the outcome is based on reliable register information. Also, at the time of the baseline data collection, technical measurements—accelerometry and electromyography—were not mainstream in the field of occupational epidemiology. Many studies in this field are either based on individuals who are still working, that is, information on retirement is based on expectations and not registers—or alternatively—is based on information from retired individuals, for which reason information about the working environment may be affected by, for example, recall bias. Further, it is a limitation that the psychosocial and physical working conditions are only measured at baseline. Participants may have changed exposure levels during the follow-up period, which could have influenced the association with early retirement. Future research could employ a study design with repeated measures of working conditions and thereby improve the assessment and reduce potential misclassification of exposure. Further, it cannot be ruled out whether jobs in some small proportion of workers would change between the time of the assessment in the surveys and the linkage of the outcome variable from the registry.

A limitation is that we used single-item questions to determine factors within the psychosocial working environment instead of multi-item scales. Still, we observed associations between these simple questions and leaving the labour market before the state pension age. This is in agreement with a previous study reporting associations between the same single-item questions as in the present study and working beyond state pension age [28]. However, not employing the entire validated scales hampers the comparison to other studies measuring psychosocial working conditions using the full scales from the COPSOQ. By contrast, subsequent use at workplaces—for example, in risk assessments—can benefit from making the questionnaire as short as possible to save time and increase the response rate.

In 2012, it was possible to resign one’s membership of VERP. If one chose to resign, his/her contribution to the program was refunded [39]. A large share of the members of VERP used this possibility [40]. We cannot rule out the possibility that some of the respondents in our sample, who were members of the program, while responding to the survey in 2005, 2008, or 2010, resigned in 2012. However, most of those who resigned in 2012, were younger than our sample, that is, younger than 55 years of age [40].

The sample size is a limitation to the study as it is difficult to detect possible statistical interactions with a relatively small sample of about 3000 participants. However, we still found some of the interactions to be statistically significant. Finally, it is a limitation that there is no information on non-response, that is, information regarding any potential demographic differences between the participants and the non-participants. Therefore, it cannot be ruled out that the study sample of 2800 older workers constitutes a selected sample, which may be subject to certain selection biases.

## 5. Conclusions

High physical work demands and poor psychosocial working conditions are factors that can push older workers out of the labour market prematurely. Our results indicate that a poor overall psychosocial work environment is a stronger push factor among workers with seated work than among those with physically demanding work.

## Figures and Tables

**Table 1:** Baseline characteristics of the study sample (*N* = 2800).

Variable	*N*	Percent
Sex		
Men	1280	45.7
Women	1520	54.3
Cohort		
DWECS 2005	879	31.4
DANES 2008	1383	49.4
DWECS 2010	538	19.2
Cohabiting		
Married	2148	76.7
Cohabiting	185	6.6
Single	467	16.7
Sector		
Public sector	1452	51.9
Private sector	1348	48.1
Occupational education		
Unskilled	618	22.1
Skilled	1253	44.8
Higher education	929	33.2
Family available income		
0–15 percentile	234	8.4
>15–50 percentile	993	35.5
>50–85 percentile	1138	40.6
>85–100 percentile	435	15.5
Weekly working hours		
≤35 h	786	28.1
35 < hours ≤ 40	1533	54.8
>40 h	481	17.2
Body Mass Index		
Underweight	32	1.1
Normal weight	1266	45.2
Overweight	1138	40.6
Obese	364	13.0
Smoking status		
Smoker	1025	36.6
Ex-smoker	1028	36.7
Never	747	26.7
Long-term sickness absence 2 years before baseline		
No	2393	85.5
Yes	407	14.5
Member of voluntary early retirement program		
No	384	13.7
Yes	2416	86.3
Physical demands at work		
No physical demands	1226	43.8
Physical demands	1574	56.2

**Table 2:** Association between physical and psychosocial work factors and early retirement. The risk ratios (RR) represent the highest value of each scale (reference: the lowest value). CI = confidence interval.

Work Factors	RR (95% CI)	
	Model 1 ^a^	Model 2 ^b^
Physical		
Higher physical demands	1.57 [1.41–1.74]	1.33 [1.19–1.48]
Psychosocial		
Overall psychosocial work environment ^c^	1.57 [1.38–1.78]	1.43 [1.26–1.61]
Low influence at work	1.48 [1.31–1.67]	1.30 [1.16–1.46]
High work pace	1.13 [0.97–1.32]	1.14 [0.98–1.31]
No time to tasks	0.99 [0.87–1.13]	1.19 [1.04–1.36]
No information about decisions at work	1.39 [1.22–1.59]	1.24 [1.09–1.41]
No information to do work well	1.46 [1.25–1.71]	1.31 [1.12–1.52]
No recognition from management	1.48 [1.39–1.69]	1.41 [1.24–1.60]
No possibilities for development	1.54 [1.34–1.77]	1.27 [1.11–1.47]
Member of voluntary early retirement program		
Yes	1.87 [1.59–2.19]	1.79 [1.53–2.10]

^a^: adjusted for age, sex, cohort. ^b^: adjusted for age, sex, cohort, cohabiting, sector, income, vocational education, working hours, access to VERP, lifestyle (BMI and smoking status), and previous sickness absence. ^c^: The ‘overall psychosocial work environment’ represents the average normalized 0–1 score of the other seven.

**Table 3:** Association between psychosocial work factors and early retirement, stratified by physical activity at work. The risk ratios (RR) represent the highest value of each scale (reference: the lowest value) (CI = confidence interval).

Work Factors	RR (95% CI) ^a^		Interaction ^b^(*p*-Value)
	Seated Work	Physical Work	
Psychosocial			
Overall psychosocial work environment ^c^	1.85 [1.49–2.29]	1.27 [1.10–1.48]	0.0039
Low influence at work	1.44 [1.16–1.77]	1.27 [1.11–1.46]	0.2116
High work pace	1.11 [0.85–1.44]	1.24 [1.05–1.47]	0.8731
No time to tasks	1.20 [0.96–1.50]	1.18 [1.01–1.39]	0.1527
No information about decisions at work	1.51 [1.20–1.90]	1.13 [0.97–1.31]	0.1017
No information to do work well	1.88 [1.45–2.44]	1.12 [0.93–1.34]	0.001
No recognition from management	2.00 [1.60–2.51]	1.19 [1.02–1.38]	0.0004
No possibilities for development	1.59 [1.22–2.06]	1.20 [1.02–1.40]	0.0713
Member of voluntary early retirement program			
Yes	1.67 [1.29–2.16]	1.85 [1.52–2.26]	0.0487

^a^: adjusted for age, sex, cohort, cohabiting, sector, income, vocational education, working hours, access to VERP, lifestyle (BMI and smoking status), and previous sickness absence. ^b^: The *p*-value of the interaction term between each psychosocial factor and physical activity at work is provided in the last column. ^c^: The ‘overall psychosocial work environment’ represents the average normalized 0–1 score of the other seven.

## Data Availability

The authors encourage collaboration and use of the data by other researchers. Researchers interested in using the data for scientific purposes should contact the project leader L.L.A., lla@nfa.dk.

## Supplementary Materials

The following are available online at https://www.mdpi.com/article/10.3390/ijerph18189817/s1, Table S1: Association between physical and psychosocial work factors and early retirement, stratified by education (unskilled, skilled, higher education), Table S2: Association between physical and psychosocial work factors and early retirement, stratified by sex (women, men).
