# Supplementary material for: Importance of the Working Environment for Early Retirement: Prospective Cohort Study with Register Follow-Up
Source: Int J Environ Res Public Health. 2021 Sep 17;18(18):9817. doi: 10.3390/ijerph18189817 (PMC8472036; doi:10.3390/ijerph18189817)
Supplement: Supplementary file 1 [file ijerph-18-09817-s001.zip › ijerph-1377081-supplementary.pdf]

International Journal of
Environmental Research and Public Health

MDPI

Supplementary Materials

Table S1. Association between physical and psychosocial work factors and early retirement, stratified by education (unskilled, skilled, higher education). The risk ratios (RR) represent the highest value of each scale (reference: the lowest value). [CI=confidence interval.].

Work Factors	RR (95% CI)[a]			Interaction[b] (P-value)
	Unskilled	Skilled	Higher education	
**Physical**				
Higher physical demands	1.28 [1.04–1.58]	1.18 [1.02–1.37]	1.79 [1.39–2.32]	0.0161
**Psychosocial**				
Overall psychosocial work environment[c]	1.19 [0.93–1.52]	1.57 [1.32–1.86]	1.42 [1.12–1.82]	0.264
Low influence at work	1.18 [0.95–1.47]	1.36 [1.16–1.59]	1.40 [1.06–1.83]	0.4762
High workpace	1.11 [0.87–1.42]	1.16 [0.94–1.43]	1.17 [0.85–1.60]	0.9851
No time to tasks	0.99 [0.76–1.29]	1.32 [1.10–1.58]	1.12 [0.85–1.60]	0.2162
No information about decisions at work	1.07 [0.85–1.35]	1.31 [1.10–1.56]	1.35 [1.02–1.79]	0.345
No information to do work well	0.94 [0.72–1.22]	1.35 [1.10–1.67]	1.93 [1.39–2.67]	0.0034
No regcognition from management	1.22 [0.96–1.55]	1.43 [1.20–1.72]	1.54 [1.19–1.99]	0.5542
No possibilities for development	1.13 [0.88–1.46]	1.31 [1.08–1.58]	1.57 [1.13–1.82]	0.2619
**Member of voluntary early retirement program**				
Yes	2.38 [1.68–3.37]	1.97 [1.48–2.62]	1.41 [1.12–1.77]	0.02

[a]: adjusted for age, sex, cohort, cohabiting, sector, income, working hours, access to VERP, lifestyle (BMI and smoking status), and previous sickness absence. [b]: The P-value of the interaction term between each work factor and education is provided in the last column. [c]: The 'overall psychosocial work environment' represents the average normalized 0–1 score of the other seven.

Table S2. Association between physical and psychosocial work factors and early retirement, stratified by sex (women, men). The risk ratios (RR) represent the highest value of each scale (reference: the lowest value). [CI=confidence interval.].

Work Factors	RR (95% CI)[a]		Interaction[b] (P-value)
	Women	Men	
**Physical**			
Higher physical demands	1.26 [1.11–1.44]	1.43 [1.18–1.73]	0.0418
**Psychosocial**			
Overall psychosocial work environment[c]	1.31 [1.13–1.52]	1.66 [1.33–2.07]	0.0257
Low influence at work	1.21 [1.05–1.40]	1.44 [1.19–1.74]	0.0406
High workpace	1.09 [0.92–1.29]	1.29 [1.01–1.64]	0.2452
No time to tasks	1.13 [0.96–1.32]	1.26 [1.00–1.57]	0.9857
No information about decisions at work	1.27 [1.09–1.49]	1.24 [1.00–1.53]	0.8995
No information to do work well	1.32 [1.10–1.59]	1.34 [1.06–1.69]	0.6634
No regcognition from management	1.29 [1.10–1.50]	1.61 [1.30–1.99]	0.0641
No possibilities for development	1.18 [1.00–1.40]	1.39 [1.10–1.76]	0.0799
**Member of voluntary early retirement program**			
Yes	1.92 [1.54–2.39]	1.65 [1.31–2.09]	0.4346

[a]: adjusted for age, cohort, cohabiting, sector, income, vocational education, working hours, access to VERP, lifestyle (BMI and smoking status), and previous sickness absence. [b]: The P-value of the interaction term between each work factor and gender is provided in the last column. [c]: The 'overall psychosocial work environment' represents the average normalized 0–1 score of the other seven.
